# Sarcopenia Diagnosis: Reliability of the Ultrasound Assessment of the Tibialis Anterior Muscle as an Alternative Evaluation Tool

**DOI:** 10.3390/diagnostics11112158

**Published:** 2021-11-21

**Authors:** Massimiliano Leigheb, Alessandro de Sire, Matteo Colangelo, Domenico Zagaria, Federico Alberto Grassi, Ottavio Rena, Patrizio Conte, Pierluigi Neri, Alessandro Carriero, Gian Mauro Sacchetti, Fabio Penna, Giuseppina Caretti, Elisabetta Ferraro

**Affiliations:** 1 Orthopaedics and Traumatology Unit, “Maggiore della Carità” Hospital, Department of Health Sciences, University of Piemonte Orientale, 28100 Novara, Italy; massimiliano.leigheb@uniupo.it (M.L.); colangelomatteo@gmail.com (M.C.); 2 Department of Medical and Surgical Sciences, University of Catanzaro “Magna Graecia”, 88100 Catanzaro, Italy; 3 Radiology Unit, “Maggiore della Carità” Hospital, Department of Translational Medicine, University of Piemonte Orientale, 28100 Novara, Italy; dzagaria19@gmail.com (D.Z.); grhxco@alice.it (P.C.); pierneri@alice.it (P.N.); alessandro.carriero@uniupo.it (A.C.); 4 Orthopaedics and Traumatology Unit, IRCCS Policlinico San Matteo, Department of Clinical-Surgical, Diagnostics and Pediatrics Sciences, University of Pavia, 27100 Pavia, Italy; federico.grassi@unipv.it; 5 Thoracic Surgery Unit, “Maggiore della Carità” Hospital, Department of Health Sciences, University of Piemonte Orientale, 28100 Novara, Italy; ottavio.rena@uniupo.it; 6 Nuclear Medicine Unit, “Maggiore della Carità” Hospital, 28100 Novara, Italy; gianmauro.sacchetti@maggioreosp.novara.it; 7 Department of Clinical and Biological Sciences, University of Torino, 10125 Torino, Italy; fabio.penna@unito.it; 8 Department of Biosciences, University of Milan, 20133 Milan, Italy; giuseppina.caretti@unimi.it; 9 Cell and Developmental Biology Unit, Department of Biology, University of Pisa, 56127 Pisa, Italy; elisabetta.ferraro@unipi.it

**Keywords:** sarcopenia, ultrasonography, muscle, muscle thickness, muscle stiffness

## Abstract

Sarcopenia is a skeletal muscle disorder characterized by reduced muscle mass, strength, and performance. Muscle ultrasound can be helpful in assessing muscle mass, quality, and architecture, and thus possibly useful for diagnosing or screening sarcopenia. The objective of this study was to evaluate the reliability of ultrasound assessment of tibialis anterior muscle in sarcopenia diagnosis. We included subjects undergoing total or partial hip replacement, comparing measures with a healthy control group. We measured the following parameters: tibialis anterior muscle thickness, echogenicity, architecture, stiffness, skeletal muscle index (SMI), hand grip strength, and sarcopenia related quality of life evaluated through the SarQoL questionnaire. We included 33 participants with a mean age of 54.97 ± 23.91 years. In the study group we found reduced tibialis anterior muscle thickness compared to the healthy control group (19.49 ± 4.92 vs. 28.94 ± 3.63 mm, *p* < 0.05) with significant correlation with SarQoL values (r = 0.80, *p* < 0.05), dynamometer hand strength (r = 0.72, *p* < 0.05) and SMI (r = 0.76, *p* < 0.05). Moreover, we found reduced stiffness (32.21 ± 12.31 vs. 27.07 ± 8.04 Kpa, *p* < 0.05). AUC measures of ROC curves were 0.89 predicting reduced muscle strength, and 0.97 predicting reduced SMI for tibialis anterior muscle thickness, while they were 0.73 and 0.85, respectively, for muscle stiffness. Our findings showed that ultrasound assessment of tibialis anterior muscle might be considered a reliable measurement tool to evaluate sarcopenia.

## 1. Introduction

Sarcopenia is currently defined as a progressive and generalized skeletal muscle disorder, characterized by a low muscle mass and function with a consequent increasing risk of falls and fractures [1]. Sarcopenia is one of the most relevant changes occurring in ageing, but it could be also related to oncologic [2], neurological [3], gastrointestinal [4,5,6], cardiovascular, and respiratory diseases [7]. Sarcopenic patients have an increased physical disability and an overall higher rate of mortality among community-dwelling older people [8]. Patients affected by sarcopenia commonly need adequate and specific follow-up and tailored rehabilitative treatment [9,10]. Sarcopenia prevalence ranges from 5 to 35%, with the highest percentage occurring among elderly people [11]; moreover, patients with sarcopenia have a higher risk of musculoskeletal disorders [12]. Therefore, we highlight the incumbent need of a standardized diagnostic tool, capable of objectively discriminating and defining sarcopenia among all groups.

The main clinical criteria of sarcopenia (reduced muscle mass, reduced muscle strength, and poor physical performance) could be used both for the diagnosis and the assessment of its severity [1]. The International Working Group on Sarcopenia (IWGS) has described as general criteria for sarcopenia evaluation a combination of poor physical performance with low muscle mass [13]. Recently, the European Working Group on Sarcopenia in Older People (EGWSOP) 2018 Update defined the occurrence of “probable sarcopenia” in individuals with low muscle strength [1]. Thus, sarcopenia is confirmed if muscle strength is associated with reduced muscle quantity or quality assessed by dual-energy X-ray absorptiometry (DXA), lumbar muscle cross-sectional area through computer tomography (CT), or magnetic resonance imaging (MRI). In case of the concomitant presence of reduced physical performance, sarcopenia is considered severe [1]. However, the EGWSOP2 criteria have been criticized by the literature, asthey can underestimate the presence of sarcopenia in males, and the strict cut-off point for muscle strength might result in underdiagnosis [14]. In this context, the SARC-F is a self-reported questionnaire considered as a useful tool to investigate frailty and poor muscle strength in common clinical practice [15]; this questionnaire showed to a low-to-moderate sensitivity and a very high specificity [16] and might be able to accurately discriminate sarcopenic from non-sarcopenic subjects [17].

As stated above, muscle quantity evaluation is crucial for the assessment in sarcopenia, and it can be estimated by a variety of techniques, with good reproducibility, and defined cut-off points [1]. On the other hand, muscle quality definition is more cryptic, as it can refer to both micro- and macroscopic changes in muscle architecture and composition, but there is a lack of standardized assessment methods in clinical practice [18]. As far the evaluation of muscle mass, DXA is considered the gold standard in measuring appendicular lean mass [19], consisting of a whole-body scan though X-rays emission. It has the advantage of being widely available, accurate, and with good reproducibility. Moreover, DXA also provides information about bone status. However, it needs proper education and formation of personnel involved in imaging acquisition [20]. CT can be used to assess both muscle mass and quality [21], since it can estimate the degree of fat infiltration. However, it has no clear or standardized cut off points, it is difficult to use in clinical practice, and utilizes large doses of radiations [22]. Furthermore, MRI might be used to evaluate both muscle quality and quantity, with high accuracy and reproducibility, identifying the potential presence of intermuscular adipose tissue [23,24,25]. However, it has no clear thresholds, is expensive, and takes longer time for images acquisition; thus, it might have some contraindications [26]. In this context, UltraSonography (US) might be useful, as it can acquire information about both muscle quantity and quality. Indeed, measurement of muscle thickness, cross-sectional area, fascicle length, pennation angle, and echogenicity have been proposed as measures in sarcopenia evaluation [27,28,29]. These parameters may be altered in older subjects, in lower limb antigravity muscles such as quadriceps femoris and gastrocnemius medialis [28]. Therefore, US assessment of tibialis anterior muscle thickness appeared to be a promising index of muscle quantity, strength, and performance, correlating both with dynamometer and physical tests [30,31,32,33]. Other characteristics have been investigated as parameters for skeletal muscle evaluation, such as altered echogenicity, which traditionally correlates with lower muscle quality and reduced strength [34], perfusion, and also muscle elasticity [35]. Interestingly, in the context of ultrasound-based methodologies, shear wave elastography (SWE) allows abnormal passive muscle stiffness detection. Abnormal stiffness has been described in neuromuscular pathologies and is often related to inflammation and higher risks of muscle damage [36,37,38]. However, to date, no study has yet proposed muscle stiffness assessment by SWE for sarcopenia definition.

Overall, despite the emergent evidence, sarcopenia assessment criteria by US are still debated, and the EGWSOP group itself encourages further research to validate prediction equations in different population [1]. Therefore, this study aimed to evaluate the reliability of US and SWE of tibialis anterior muscle in the sarcopenia assessment compared to gold standard diagnostic tests.

## 2. Methods

### 2.1. Participants

We excluded patients with: (a) terminal illness; (b) acute or chronic neuromuscular diseases; (c) severe cognitive impairment; (d) NYHA class 3–4 heart failure; (e) renal failure; (f) cirrhosis; (g) pulmonary emphysema; (h) chronic obstructive pulmonary disease; (i) pregnancy; (j) diabetes; (k) chronic inflammatory diseases.

In this observational study, we included two groups of patients: (1) potentially sarcopenic patients; (2) healthy controls. The potentially sarcopenic patients were subjects aged 65 years or more, who underwent total or partial hip arthroplasty surgery due to femoral fracture or hip osteoarthritis, referring to the Orthopedic Trauma Service of “Maggiore della Carità” University Hospital, Novara, Italy, between November 2019 and December 2020. We also included subjects aged between 18 and 40 years, without any previous or incident pathologies, as healthy controls.

The Ethics Committee of Novara (Italy) approved the study (protocol number 62/18). All participants were asked to carefully read and sign an informed consent. Researchers protected the participants’ privacy, and all the procedures were conducted according to the principles of the Declaration of Helsinki.

### 2.2. Outcomes

Demographic, anamnestic, clinical characteristics, and medical imaging findings were collected in all patients. We also administered and recorded the SarQoL, a self-reported questionnaire consisting of 22 questions encompassing 7 domains and 55 items. The domains were divided into: (a) “Physical and Mental Health”; (b) “Locomotion”; (c) “Body Composition”; (d) “Functionality”; (e) “Activities of Daily Living”; (f) “Leisure Activities”; (g) “Fears”. Results were presented as numerical scores between 0 and 100, where higher values indicate better QoL in subjects with sarcopenia [39].

Then, we assessed the skeletal muscle index (SMI), adjusting the absolute level of appendicular skeletal muscle mass (in kg) with height squared (kg/m^2^), was assessed by the DXA, the gold standard for sarcopenia diagnosis [40].

Muscle strength was evaluated by the hand grip strength test (HGS), through the hand-held dynamometer (Jamar hydraulic hand dynamometer, Sammons Preston, Bolingbrook, IL, USA), considering the maximum value (in kilograms) of three consecutive measurements of the upper dominant limb (with a pause of 1 min after each measurement); values below 27 kg for men and 16 kg for women indicate reduced muscle strength and probable sarcopenia [41].

Then, the sarcopenia diagnosis was performed according to the EGWSOP2 criteria [1] in all the study cohort.

Furthermore, all patients underwent US B-mode and SWE evaluations with a Toshiba Aplio 500 ultrasound device (Toshiba Medical Systems, Tokyo, Japan). The PLT1005BT linear transducer with frequency range 7.0–14.0 MHz was used. The measurements were carried out on the right leg of each patient in full extension, resting in supine position, advising to not exercise in the 30 min before investigation. Tibialis anterior muscle was evaluated at proximal 30% between the popliteal crease and tip of the lateral malleolus [29].

**Figure 1:**
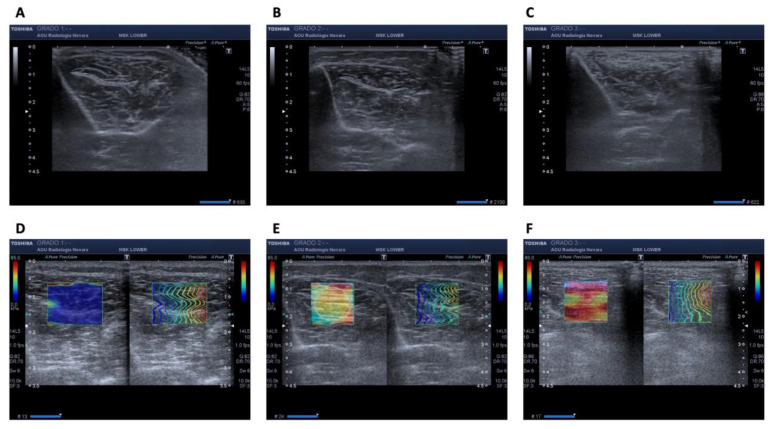
Tibialis anterior muscle ultrasound assessment in terms of echogenicity, grade 1 (**A**), grade 2 (**B**), and grade 3 (**C**), and stiffness, measured with shear wave elastography with grade 1 (**D**), grade 2 (**E**), and grade 3 (**F**).

All the exams were performed by the same operator with expertise in musculoskeletal US, under the same environmental conditions, and ultrasonographic scans were performed transversely to the muscle. Echogenicity, architecture and SWE were graded according to a previously published technique [42] and explained as follow. We collected: (a) tibialis anterior muscle thickness, measured in millimeters, as primary outcome; (b) muscle echogenicity, identified with a gray scale, where 0 indicated normality, 1 slightly increased echogenicity compared to the surrounding structures, and 2 marked increase in echogenicity; (c) muscle architecture of the same muscle, identified with a scale where 0 indicated that intramuscular fibers were clearly visible and pinnation angle easily identified, 1 in which these structures were only partially identifiable, and 2 where the original muscular architecture was no longer identifiable; (d) muscle stiffness of the proximal third of the tibialis anterior muscle, with SWE technique, measured both in Kpa and on a color scale (with blue color indicating minor stiffness and red color indicating higher stiffness), with a grade 0 in which the blue color absolutely prevailed, a grade 1 where more than half of the examined structure was blue, and a grade 2 in which most of the area of the region of interest was yellow-red (see Figure 1).

### 2.3. Statistical Analysis

Statistical analysis was performed using STATA v.13 (StataCorp LP, College Station, Texas, TX, USA). The continuous variables are presented as means ± standard deviations, medians, and interquartile, whereas categorical data are expressed as counts and percentages.

The strength of correlations between the variables of interest was assessed using the Pearson’s linear correlation coefficient. The predictive models were evaluated utilizing ROC curves and logistic models. For the classification of discriminatory power by the AUC curve [43], values >0.7 and ≤0.9 were considered as excellent discriminatory power. Only *p* values lower than 0.05 were considered statistically significant.

## 3. Results

Out of 43 patients that were pre-screened as eligible for this study, 33 participants were included (16 male and 17 female; mean age: 54.97 ± 23.91 years old). They were divided into two groups: (1) 18 potentially sarcopenic elderly subjects (11 male and 7 female) who had undergone total or partial hip arthroplasty surgery, mean aged 75.55 ± 8.54; (2) 15 healthy controls (5 male and 10 female), mean aged 30.27 ± 4.45.

The body mass index (BMI) was found higher, yet not significant, in group 1, compared to healthy controls (26.71 ± 3.90 vs. 23.73 ± 3.07 kg/m^2^). The average values deriving from SarQoL were lower in the group 1 compared to group 2 (53.78 ± 14.92 vs. 99.34 ± 1.02, *p* < 0.05).

Muscle strength test at the HGS showed lower values in group 1 compared to group 2 (21.22 ± 13.48 vs. 48.46 ± 13.32 kg, *p* < 0.05); tibialis anterior muscle thickness was 19.49 ± 4.92 mm in group 1 compared to 28.94 ± 3.63 mm in group 2 (*p* < 0.05) while muscle stiffness was 32.21 ± 12.31 Kpa in group 1 compared to 27.07 ± 8.04 Kpa in group 2 (*p* < 0.05). SMI was also measured in the potentially sarcopenic patients by DXA (6.52 ± 1.29 kg/m^2^) (see Table 1 for further details).

Tibialis anterior muscle thickness was significantly correlated to SarQoL values (r = 0.80, *p* < 0.05); dynamometer hand strength (r = 0.72, *p* < 0.05); and SMI evaluated by DXA (r = 0.76, *p* < 0.05).

From a broad perspective, we found reduced muscle strength in eight (24.2%) patients, and reduced muscle mass in seven (21.2%) patients. These seven patients were diagnosed with sarcopenia according to the EGWSOP2 criteria [1], and they all belonged to group 1. Among patients with reduced muscle strength, four (50%) patients showed an increased echogenicity (grade 2), six (75%) showed a markedly compromised architecture (grade 2), and four (50%) an increased muscle stiffness (grade 2) at SWE. Among patients with reduced muscle mass, six (85.7%) patients showed markedly increased echogenicity (grade 2), six (85.7%) showed a markedly compromised architecture (grade 2), and four (57.1%) an increased muscle stiffness (grade 2) at SWE.

**Figure 2:**
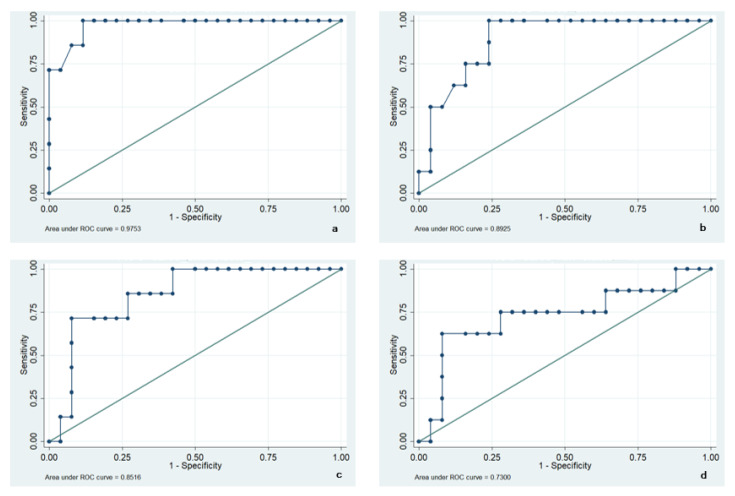
ROC curves of tibialis anterior muscle thickness with SMI (**a**) and HGS (**b**); ROC curves of tibialis anterior muscle stiffness with SMI (**c**) and HGS (**d**).

Lastly, we measured the AUC (area under curve) of ROC curves for tibialis anterior to evaluate if tibialis anterior muscle thickness obtained by US and muscle stiffness obtained by SWE might be potentially able to diagnose sarcopenia. AUC of ROC curves were 0.89 compared to reduced muscle strength, and 0.97 compared to SMI. Regarding muscle stiffness, we found an AUC of 0.73 and 0.85, respectively. Since AUC values >0.7 and ≤0.9 were considered as excellent discriminatory power, we can conclude that the addition of the evaluation of the muscle thickness but also of the muscle stiffness might better provide a diagnosis of sarcopenia (see Figure 2).

## 4. Discussion

The use of skeletal muscle ultrasound has recently been expanded in clinical practice to support the diagnosis of sarcopenia. In fact, US B-mode and SWE should be considered as low-cost diagnostic tests, transportable to the patient’s bedside, and radiation sparing compared to gold standard techniques (i.e., DXA, CT, and MRI) [1].

The SARCUS (SARCopenia through UltraSound) group has indeed proposed consensus for anatomical landmarks and measurement standardization [29], considering several muscle characteristics, as muscle thickness, pennation angle, fascicle length, echo-intensity, and cross-sectional area. Although ultrasound assessment might be useful for detecting the loss of muscle mass and muscle quality alteration in patients, a high degree of standardization in ultrasound protocols is necessary [28].

In the present observational study, we found that tibialis anterior muscle thickness measured at proximal 30% between the popliteal crease and tip of the lateral malleolus, is significantly and strongly correlated with SarQoL values, dynamometer hand strength, and SMI, in a cohort of hip fracture patients; these osteoporotic subjects are commonly at high risk of developing sarcopenia and should be adequately assessed and treated [43,44,45]. We also found that altered muscle architecture, echogenicity, and stiffness at tibialis anterior level are frequently associated with muscle mass and muscle strength reduction. Moreover, tibialis anterior muscle thickness and stiffness measured in Kpa showed excellent discriminatory power in prediction of muscle mass and strength reduction (evaluated by ROC curves) compared to dynamometer and DXA measurements, Finally, comparing muscle thickness evaluation and stiffness at the same landmark, the first seems to be more reliable in identifying patients with reduced muscle mass and function.

In the literature, several studies have compared ultrasound assessment to DXA in muscle mass measurement. Ismail et al. [46] found that ultrasound morphometry values are associated with lean body mass and strength, in community-dwelling female subjects; however, they evaluated primarily rectus femoris muscle characteristics. Further, Berger et al. underlined a good concordance between rectus femoris ultrasound thickness and DXA lean mass assessment in older community dwelling people [47]. Another study [48] compared ultrasound assessment of anterior and posterior aspects of the thigh with lean mass evaluated by DXA in middle-aged and older adults, showing a significant correlation. Moreover, a recent study suggested ultrasonographic muscle thickness of tibialis anterior as DXA alternative in evaluating muscle mass of stroke survivors [49].

Whereas ultrasound assessment of lower limb muscle, such as rectus femoris muscle [50] and gastrocnemius [51] thickness, has been widely examined in literature, even with proposed cut-off measures, upper limb has been less considered, as its volumetric alterations might be more age-dependent [52]. Finally, a recent study investigated the potential predictive value of geniohyoid muscle in sarcopenic patients, with good results [53]. As for muscle architecture, it has been correlated with muscle mass and performance reduction [54]; although, the comparison with muscle thickness evaluation seems less reliable and more user dependent. On the other hand, SWE was suggested for staging chronic diseases, determining therapeutic response, and monitoring age-related changes, including sarcopenia and clinical frailty syndrome [55,56]. Furthermore, it has been utilized to assess skeletal muscle spasticity in post stroke patients [57,58].

However, this is the first study, to our knowledge, that proposes employing SWE of the tibialis anterior muscle as a sarcopenia diagnostic tool. Our data suggest that muscle ultrasonography and SWE at tibialis anterior muscle might be reliable tools compared to gold standard diagnostic tests and examinations to discriminate patients with reduced muscle mass and function and diagnose sarcopenia in the general population.

Finally, we are aware that the present study is not free from limitations: first, the lack of a comparable control group in terms of age; second, the absence of analysis of the potential influence that comorbidities might have on muscle stiffness; and lastly, the monocentric study design and the small sample size might not guarantee high external validity as in large multicentric studies.

## 5. Conclusions

Taken together, our findings showed that ultrasound assessment of tibialis anterior muscle might be a reliable tool to measure muscle quantity and quality in the diagnosis of sarcopenia. However, albeit muscle thickness and stiffness at this location might have considerable discriminating capacities, further studies are warranted to generalize these findings and to better evaluate comparison with other muscles in terms of diagnostic potential, as well as possible cut-off values to ensure an affordable sarcopenia diagnosis in clinical practice.

## Figures and Tables

**Table 1:** Outcomes in potentially sarcopenic patients (group 1) and healthy controls (group 2).

	Group 1 (n = 18)	Group 2 (n = 15)	*p* Value
Age (years)	75.55 ± 8.54	30.27 ± 4.45	<0.05
BMI (kg/m^2^)	26.71 ± 3.90	23.73 ± 3.07	<0.05
TA thickness (mm)	19.49 ± 4.91	28.94 ± 3.63	<0.05
TA stiffness (Kpa)	32.21 ± 12.32	27.07 ± 8.04	<0.05
HGS (kg)	21.22 ± 13.47	48.47 ± 13.32	<0.05
SarQoL	53.78 ±14.92	99.34 ± 1.02	<0.05

Values are expressed as means ± standard deviations. Statistical analysis was performed through ANOVA test. Abbreviations: SarQoL: Sarcopenia Quality-of-Life questionnaire; HGS: Hand Grip Strength; TA: Tibialis Anterior muscle.

## Data Availability

Dataset is available on request.
